# Non-neural tyrosine hydroxylase, via modulation of endocrine pancreatic precursors, is required for normal development of beta cells in the mouse pancreas

**DOI:** 10.1007/s00125-014-3341-6

**Published:** 2014-08-01

**Authors:** Patricia Vázquez, Ana M. Robles, Flora de Pablo, Catalina Hernández-Sánchez

**Affiliations:** 1 3D (Development, Differentiation, Degeneration) Lab, Department of Cellular and Molecular Medicine, Centro de Investigaciones Biológicas (CSIC), Ramiro de Maeztu 9, 28040 Madrid, Spain; 2 Centro de Investigación Biomédica en Red de Diabetes y Enfermedades Metabólicas Asociadas (CIBERDEM) (ISCIII), Ministerio de Economía y Competitividad, Spain, http://www.ciberdem.org/

**Keywords:** Beta cells, Catecholamines, Dopamine, Glucagon, Insulin, Islet development, Neurogenin 3, Tyrosine hydroxylase

## Abstract

**Aims/hypothesis:**

Apart from transcription factors, little is known about the molecules that modulate the proliferation and differentiation of pancreatic endocrine cells. The early expression of tyrosine hydroxylase (TH) in a subset of glucagon^+^ cells led us to investigate whether catecholamines have a role in beta cell development.

**Methods:**

We studied the immunohistochemical characteristics of TH-expressing cells in wild-type (*Th* ^*+/+*^) mice during early pancreas development, and analysed the endocrine pancreas phenotype of TH-deficient (*Th* ^*−/−*^) mice. We also studied the effect of dopamine addition and TH-inhibition on insulin-producing cells in explant cultures.

**Results:**

In the mouse pancreas at embryonic day (E)12.5–E13.5, the ∼10% of early glucagon^+^ cells that co-expressed TH rarely proliferated and did not express the precursor marker neurogenin 3 at E13.5. The number of insulin^+^ cells in the *Th* ^*−/−*^ embryonic pancreas was decreased as compared with wild-type embryos at E13.5. While no changes in pancreatic and duodenal homeobox 1 (PDX1)^+^-progenitor cell number were observed between groups at E12.5, the number of neurogenin 3 and NK2 homeobox 2 (NKX2.2)-expressing cells was reduced in *Th* ^*−/−*^ embryonic pancreas, an effect that occurred in parallel with increased expression of the transcriptional repressor *Hes1*. The potential role of dopamine as a pro-beta cell stimulus was tested by treating pancreas explants with this catecholamine, which resulted in an increase in total insulin content and insulin^+^ cells relative to control explants.

**Conclusions/interpretation:**

A non-neural catecholaminergic pathway appears to modulate the pancreatic endocrine precursor and insulin producing cell neogenesis. This finding may have important implications for approaches seeking to promote the generation of beta cells to treat diabetes.

**Electronic supplementary material:**

The online version of this article (doi:10.1007/s00125-014-3341-6) contains peer-reviewed but unedited supplementary material, which is available to authorised users.

## Introduction

Studies of developmental biology have led to significant advances in our understanding of the generation of insulin-producing cells [1, 2]. However, further research is required to unravel the molecular mechanisms that underlie the processes of proliferation, differentiation and survival, which maintain the pool of pancreatic progenitor cells and lead to a mature beta cell phenotype. These molecules include specific combinations of well-characterised transcription factors [3, 4] and less well known secreted signals [5–7]. In the present study, we set out to investigate the putative role of the catecholaminergic pathway in beta cell development.

Pancreas morphogenesis involves two overlapping phases. The primary transition, from embryonic day (E) 8.5/9 to E12.5, encompasses the specification of the pancreatic epithelium by the expression of PDX1 and PTF1A [8–10], the protrusion of the pancreatic epithelium into the surrounding mesenchyme and the beginning of expansion and branching. The secondary transition, from E13.5 to E15.5, involves massive differentiation of beta cells and continued expansion and branching [11]. The initial stages of foregut endoderm pancreatic specification and epithelial expansion are strongly influenced by signals from neighbouring tissues, including the mesenchyme [5, 11, 12]. Growth factors known to influence early pancreatic cell decisions include fibroblast growth factors, Wnt and TGFβ family members [5, 6].

At E12.5, the pancreatic epithelium in the mouse is composed mainly of multipotent pancreatic progenitors that express PDX1 and some early differentiated endocrine cells containing glucagon [13]. The PDX1^+^ progenitors are progressively committed to more restricted fates by the combinatorial expression of several transcription factors [14]. The activation of neurogenin 3 (NGN3) expression in these precursors triggers the specific gene regulatory networks that define the endocrine programme [15, 16]. NGN3 plays an essential role at this stage, as evidenced by the impaired development of islets in NGN3-deficient mice [15]. *Nkx2.2,* one of the genes involved in this cascade, acts downstream of NGN3 and is required for initial beta cell differentiation and, to a lesser extent, for the differentiation of other endocrine cells [17]. Notch signalling also greatly influences the transition from a multipotent progenitor towards an endocrine precursor [18, 19]. HES1, a downstream effector of the Notch pathway, represses the transcriptional activity of the *Neurog3* gene (which encodes NGN3), thus preventing premature endocrine differentiation [20, 21].

In addition to the complex intrinsic network of pancreatic transcription factors, extrinsic signals act as modulators of cell progenitor generation and lineage or fate decisions [7, 11]. No function has been ascribed to catecholamines in pancreatic development, although the pioneering studies of Teitelman and Lee [22] demonstrated the presence of a small subpopulation of cells expressing tyrosine hydroxylase (TH), the first enzyme of the catecholaminergic pathway, in the mouse pancreatic bud by E10. Catecholamines have recently been implicated in adult neurogenesis [23] and embryonic haematopoiesis [24]. Moreover, we have previously demonstrated that TH is required for heart morphogenesis and cardiomyocyte differentiation [25], broadening the spectrum of neurohormonal effects of catecholamines to other functions in development and differentiation. The aim of this study was to investigate a possible novel role of catecholamines in pancreatic development.

## Methods

Detailed methods, primer and probe sequences, and antibodies used are provided in the electronic supplementary material (ESM) Methods and ESM Tables 1–3. The sources of chemical substances are provided in ESM Table 4.

### Mice and embryos

All procedures involving animals were approved by the ethics committee of Centro de Investigaciones Biológicas and were in accordance with the European Union guidelines. The C57BL6/J TH heterozygote mouse strain was kindly provided by R. D. Palmiter (University of Washington, Seattle, WA, USA) [26], and was backcrossed with wild-type CD1 mice for up to ten generations (for further details see the ESM Methods).

### Immunoblotting

Pancreas samples of the indicated ages were pooled, homogenised and analysed by immunoblotting using standard procedures (for further details, see the ESM Methods).

### Pancreas explant cultures

E13.5 dorsal pancreatic buds were cultured on coverglasses coated with 25 mg/l collagen, in 24-well plates containing 1 ml of DMEM with 10% (vol./vol.) FBS, 1% (vol./vol.) penicillin/streptomycin and 1% (vol./vol.) glutamine. Where indicated, the cultured medium was supplemented with 0.04 mmol/l dopamine or 1 mmol/l α-methyl-l-tyrosine. Explants were cultured for up to 5 days (after 24 h of stabilisation) at 37°C and 5% CO_2_, and the medium was refreshed daily. For cell proliferation experiments, explants were treated with 5 μmol/l BrdU. After culture, the explants were processed for whole mount, tissue section or cytospin (for further details, see the ESM Methods).

### Immunohistochemistry and TUNEL

E12.5 and E13.5 embryos were fixed overnight at 4°C with 4% (wt/vol.) PFA, embedded in paraffin, immunostained and TUNEL analysed using standard procedures (for further details, see the ESM Methods).

### RNA isolation and quantitative real-time PCR

Total RNA from pancreas was extracted using TRIzol Reagent, and reverse transcription performed with random primers and Superscript III enzyme (all from Life Technologies, Carlsbad, CA, USA) according to the manufacturer’s instructions. Quantitative real-time PCR was performed in a 7900 HT-Fast real-time PCR (Life Technologies) system with Taqman Universal PCR Master Mix using Taqman assays (Life Technologies) or probes from the Universal Probe Library (Roche, Mannheim, Germany).

### In vivo BrdU labelling

Pregnant mothers were i.p. injected with BrdU (100 mg/kg body weight) 2 h before sacrifice. For details of BrdU detection and cell counting, see the ESM Methods.

### ELISA

Catecholamines were determined by ELISA (for further details, see the ESM Methods).

### Images and statistical analysis

Images were collected by confocal microscopy (Leica TCS-SP5; Leica Microsystems, Wetzlar, Germany). For morphometric analysis, quantification of the total and epithelial pancreatic area was performed in E-cadherin-stained sections using Image J 1.48v (http://imagej.nih.gov/ij). The number of cells expressing a specific marker was determined as described in the ESM Methods.

Statistical analyses were performed using non-parametric tests (Mann–Whitney *U* test) for non-normally distributed data using GraphPad Prism 5, version 5.01 (www.graphpad.com). Data from at least three independent experiments were analysed in each case, with p values <0.05 considered significant.

## Results

### TH is expressed in a subset of early glucagon^+^ cells in the developing mouse pancreas

**Fig. 1:**
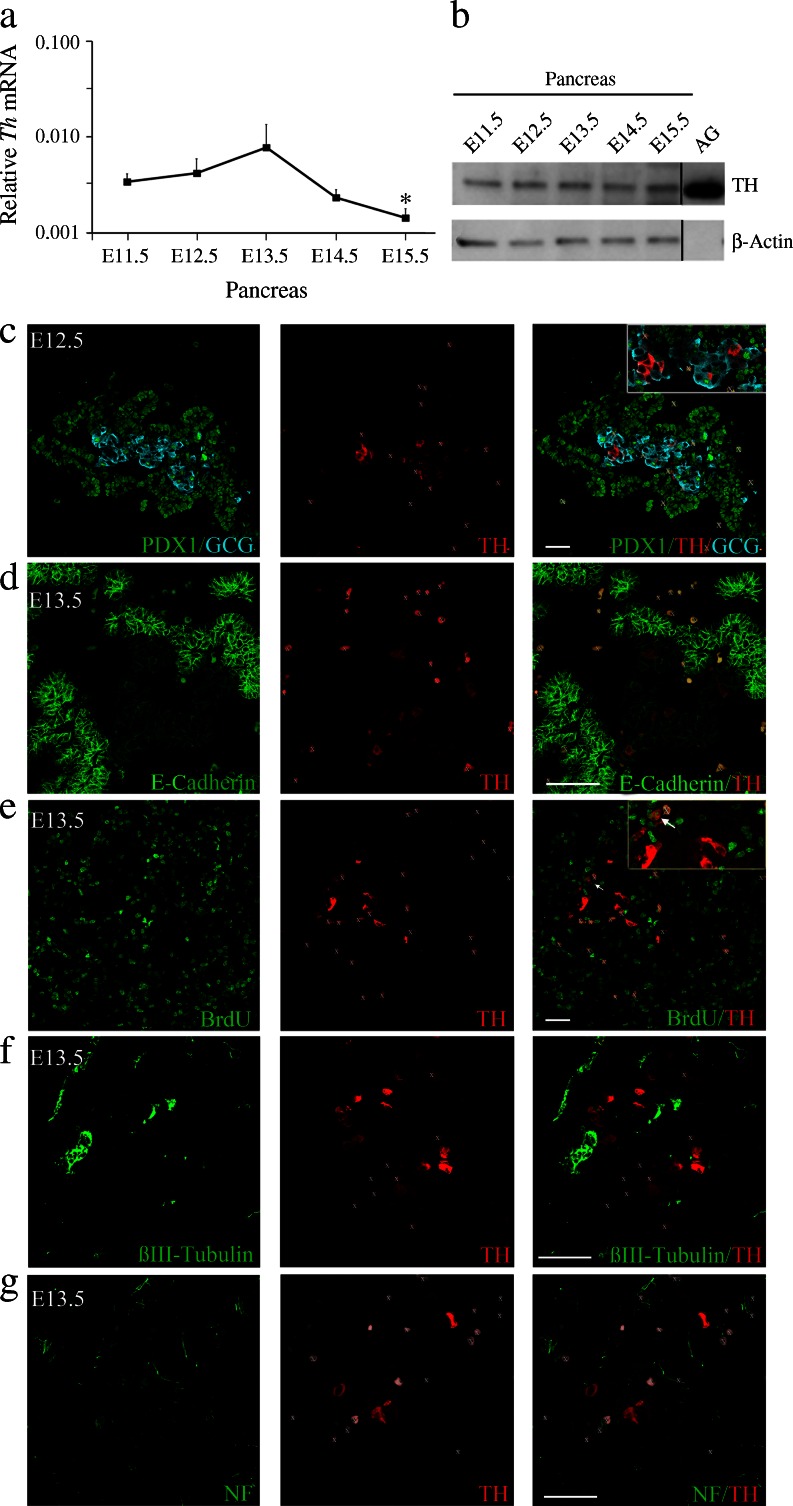
TH is expressed during early pancreas development in mice. (**a**) Quantitative real-time PCR of three to five pooled embryonic pancreases (E11.5 to E15.5). Levels of *Th* transcripts were normalised to 18S rRNA levels and the data are presented on a logarithmic scale. Results represent the mean ± SEM of at least three different pools for each embryonic stage. *p < 0.05 vs E11.5. (**b**) TH immunoblot of protein extracts from four pooled pancreases (E11.5 to E15.5). Adult adrenal gland (AG) extract was included as a positive control and β-actin as a loading control. As TH is highly expressed in AG, only low amounts of AG protein were loaded, so AG β-actin was not detected. The blot is representative of three independent experiments. (**c**–**g**) Embryonic pancreatic sections. (**c**) Co-immunostaining for PDX1 (green), TH-rabbit (red) and glucagon (GCG, cyan). (**d**) Double immunostaining for E-cadherin (green) and TH-rabbit (red). (**e**) Double-immunostaining for BrdU (green) and TH-rabbit (red). Arrows indicate double-positive cells. (**f**) Double immunostaining for βIII-tubulin (green) and TH-mouse (red). (**g**) Double-immunostaining for neurofilament (NF, green) and TH-rabbit (red). White X indicates blood cells. Scale bar, 50 μm

We first sought to better characterise the early TH-expressing cell population. Pancreatic *Th* mRNA was analysed in samples from E11.5 to E15.5, the latter being the embryonic stage during which significant sympathetic fibre innervation begins. Levels of *Th* mRNA were similar during the primary transition (E11.5–12.5) and the beginning of the secondary transition (E13.5–14.5), but decreased significantly at E15.5 (Fig. 1a), coinciding with the maximum expression of *Neurog3* and around the peak of beta cell differentiation. Immunoblotting of pancreas protein extracts revealed a 60 kDa band corresponding to TH (Fig. 1b). The band specificity was confirmed by the absence of signal in pancreatic extracts from TH-deficient embryos (ESM Fig. 1). TH protein levels were similar up to E15.5. The apparent discrepancy between the mRNA and protein profiles for TH could be due, at least in part, to the contribution of the sympathetic projections to the total content of TH protein.

Using co-immunofluorescence we further characterised the population of TH-expressing cells. Glucagon-expressing cells are one of the first pancreatic cells to differentiate. At E12.5 (Fig. 1c) and E13.5, most TH-expressing cells (∼90%) co-expressed glucagon, although TH^+^ cells represented only a small proportion (∼10%) of these early glucagon^+^ cells. At later stages of development (E14.5 and E15.5), the degree of co-expression tended to decrease (ESM Fig. 2a, b).

The TH^+^ cells shared features with the overall early glucagon population: they showed little co-localisation with PDX1 [9, 19] (Fig. 1c); they localised in clusters within regions of the pancreatic epithelium with low E-cadherin expression [27] (Fig. 1d); and rarely incorporated the mitotic marker BrdU (∼6%; Fig. 1e). These features are characteristics of hormone-expressing differentiated cells. Furthermore, we observed no co-expression of TH and the endocrine precursor marker NGN3 at E13.5 (see below).

TH^+^ cells did not express βIII-tubulin or neurofilament (neuronal markers) (Fig. 1f, g). They expressed neither SRY (sex determining region Y)-box 10 (SOX10) nor paired-like homeobox 2b (PHOX2b)—both neural crest cell [NCC] markers (ESM Fig. 3a)—confirming their non-neural phenotype. Moreover, at later stages of development (E14.5–15.5) when the first sympathetic projections (TH^+^ and βIII-tubulin^+^) were detected, the TH^+^ cell bodies did not express βIII-tubulin (ESM Fig. 2c, d). Taken together, these results demonstrate the endocrine character of early TH^+^ cells.

### Beta cell number is modulated by the catecholaminergic pathway

**Fig. 2:**
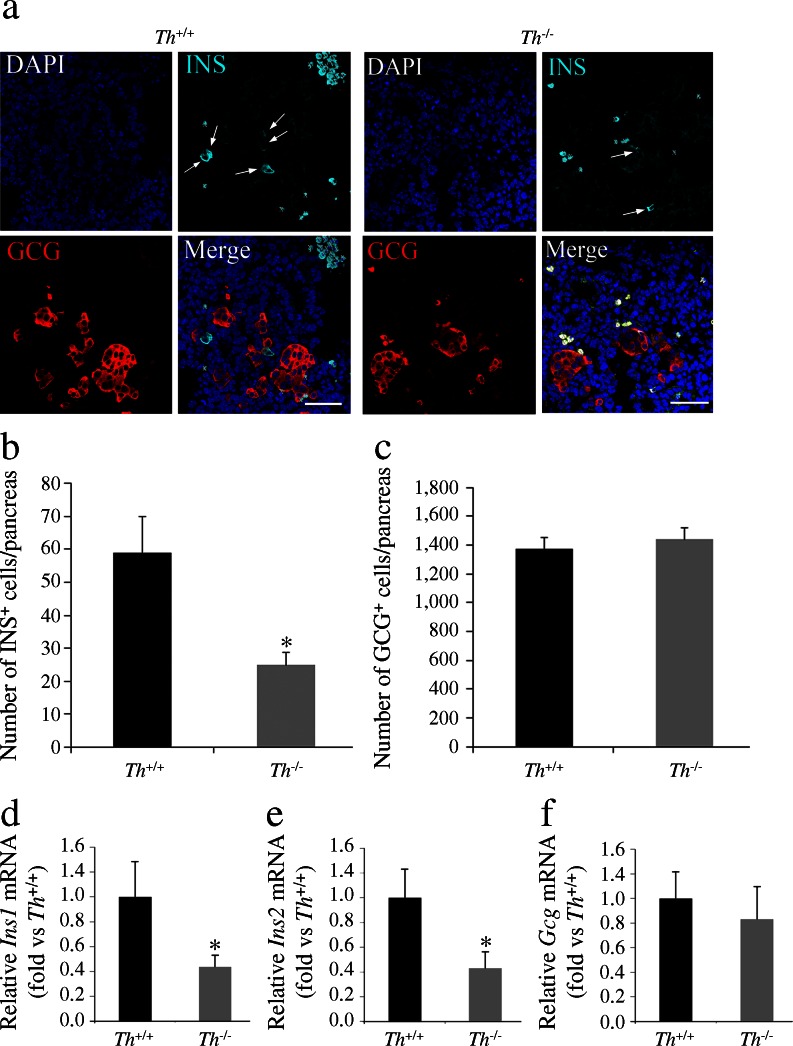
The number of insulin-expressing cells is decreased in the pancreas of TH-deficient E13.5 embryos. (**a**) Double immunostaining for insulin (INS, cyan) and glucagon (GCG, red). Nuclei are stained with DAPI. Arrows and white X indicate insulin^+^ and blood cells, respectively. Scale bar, 50 μm. (**b**, **c**) Quantification of the total number of insulin- and glucagon-expressing cells in whole pancreas. (**d**–**f**) Quantitative real-time PCR of individual pancreases. Levels of the *Ins1*, *Ins2* and *Gcg* transcripts were normalised to 18S rRNA and *Th* ^*+/+*^ values were set at 1*.* Results represent the mean ± SEM of at least five animals.*p < 0.05 vs *Th* ^*+/+*^

To date, no clear role has been ascribed to the early glucagon^+^ cells. Indeed, it has been suggested that this cell population disappears during development and does not measurably contribute to the adult organ [28, 29]. To assess the role of TH in a subpopulation of the early glucagon^+^ cells during pancreas development we phenotypically characterised the pancreas of TH-deficient mice. As embryonic lethality begins to occur from E12.5 in *Th* ^***−****/****−***^ embryos [26], we studied E13.5 embryos, the latest embryonic stage at which a significant number of live *Th* ^***−****/****−***^ embryos can be collected. Morphometric analysis of the E12.5 and 13.5 pancreatic primordia by E-cadherin immunostaining revealed no significant differences in either the epithelial area or total (epithelium + mesenchyme) pancreatic area between *Th* ^*+/+*^ and *Th* ^***−****/****−***^ embryos (data not shown). At the beginning of the secondary transition, insulin-expressing cells begin to accumulate and the population increases exponentially until E15.5. We investigated whether the emerging population of insulin-expressing cells was affected by the absence of TH expression. The number of insulin^+^ cells in the whole pancreas of E13.5 *Th* ^***−****/****−***^ embryos was decreased relative to that in the whole pancreas of *Th* ^*+/+*^ embryos. By contrast, we observed no differences between genotypes in the number of glucagon^+^ cells (Fig. 2a–c). Quantitative real-time PCR analysis confirmed lower expression levels of both insulin genes (*Ins1* and *Ins2*) in the E13.5 *Th* ^***−****/****−***^ pancreatic primordium, without changes in *Gcg* mRNA levels (Fig. 2d–f).

**Fig. 3:**
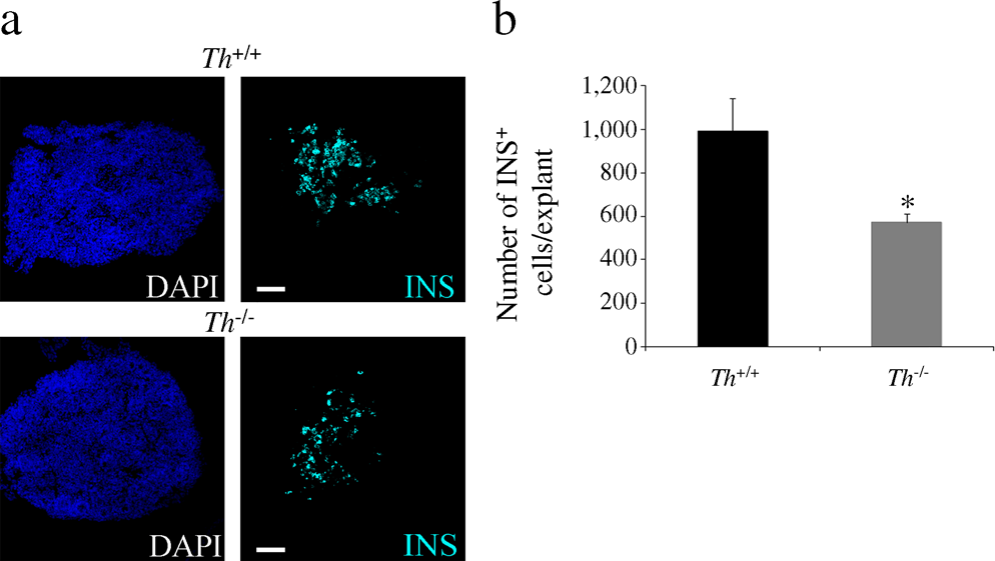
Decreased number of insulin-expressing cells in the TH-deficient pancreatic explants. (**a**) Immunostaining for insulin (INS, cyan) in E13.5 pancreatic explants cultured for 5 days. Nuclei are stained with DAPI. Scale bar, 100 μm. (**b**) Quantification of total number of insulin-expressing cells. Results represent the mean ± SEM of at least four explants per genotype. *p < 0.05 vs *Th* ^*+/+*^

To confirm the impact of TH deficiency on the final number of insulin-expressing cells, we established an organotypic pancreas culture in which the mesenchyme was preserved. Beginning at E13.5, after 24 h of stabilisation of the culture (day 0), the pancreatic explants were cultured for 5 days. The total number of insulin^+^ cells in the *Th* ^***−****/****−***^ explants at the end of the culture period was decreased compared with that in the *Th* ^*+/+*^ explants (Fig. 3a, b).

**Fig. 4:**
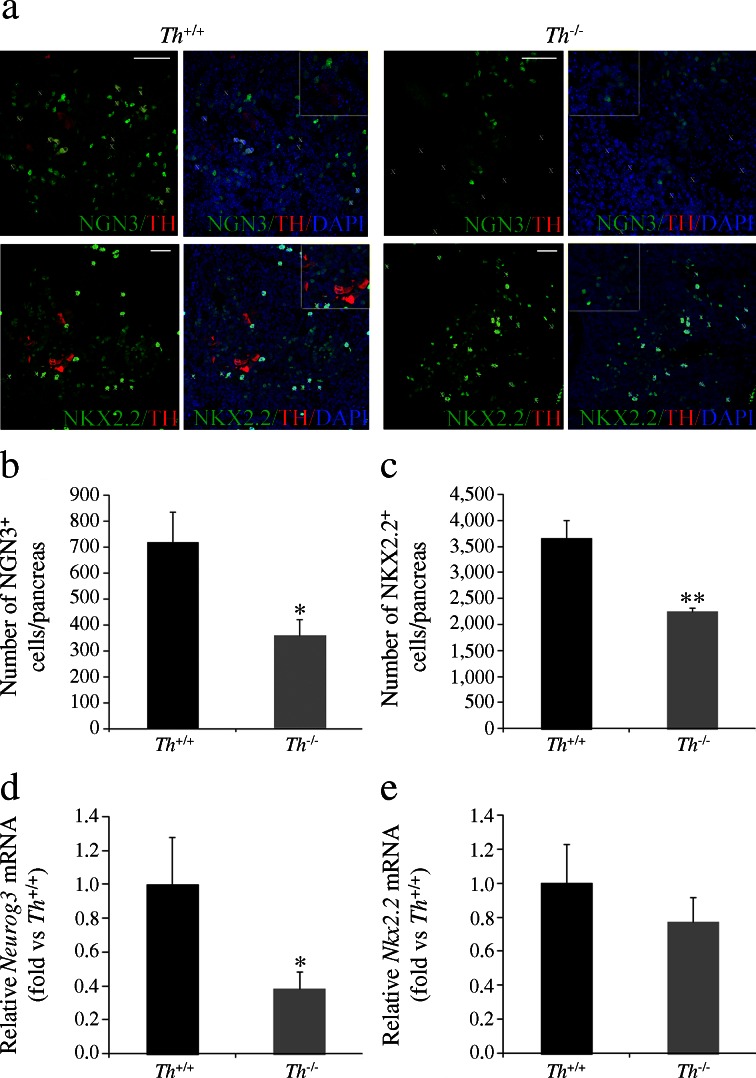
The endocrine precursor cell population is reduced in the pancreas of E13.5 TH-deficient embryos. (**a**) Double immunostaining for NKX2.2 (green), NGN3 (green) and TH-rabbit (red). Nuclei are stained with DAPI. White X indicates blood cells. The insets correspond to magnification of representative cells. Scale bar, 50 μm. (**b**, **c**) Quantification of the total number of NGN3- and NKX2.2-expressing cells in whole pancreas. (**d**, **e**) Quantitative real-time PCR of individual pancreases. Levels of *Neurog3* and *Nkx2.2* transcripts were normalised to 18S rRNA and *Th* ^*+/+*^ values were set at 1*.* Results represent the mean ± SEM of at least five embryos.* *p* < 0.05, ** *p* < 0.01 vs *Th* ^*+/+*^

Lineage studies have established that all endocrine cells are derived from NGN3-expressing progenitors [16]. Furthermore, the number of NGN3^+^ precursors during embryonic development determines the number of endocrine cells at birth [30]. We therefore analysed the NGN3^+^ cell population. We observed a decrease in the number of NGN3-expressing cells in the E13.5 *Th* ^***−****/****−***^ vs *Th* ^*+/+*^ pancreas (Fig. 4a, b). In agreement with this finding, *Neurog3* mRNA levels in the pancreas were lower in mutant embryos than in *Th* ^*+/+*^ littermates (Fig. 4d). NKX2.2 is a transcription factor required for the determination of beta cell fate [17], and its expression is regulated by NGN3 [31]. We found that the number of cells expressing NKX2.2 in the *Th* ^***−****/****−***^ pancreas was decreased compared with that in the *Th* ^*+/+*^ littermates (Fig. 4a, c). A similar trend was observed for *Nkx2.2* mRNA levels, although the differences between the *Th* ^*+/+*^ and *Th* ^***−****/****−***^ groups did not reach statistical significance (Fig. 4e).

### The pancreatic phenotype of TH-deficient mice involves an increase in Hes1 expression before secondary transition

**Fig. 5:**
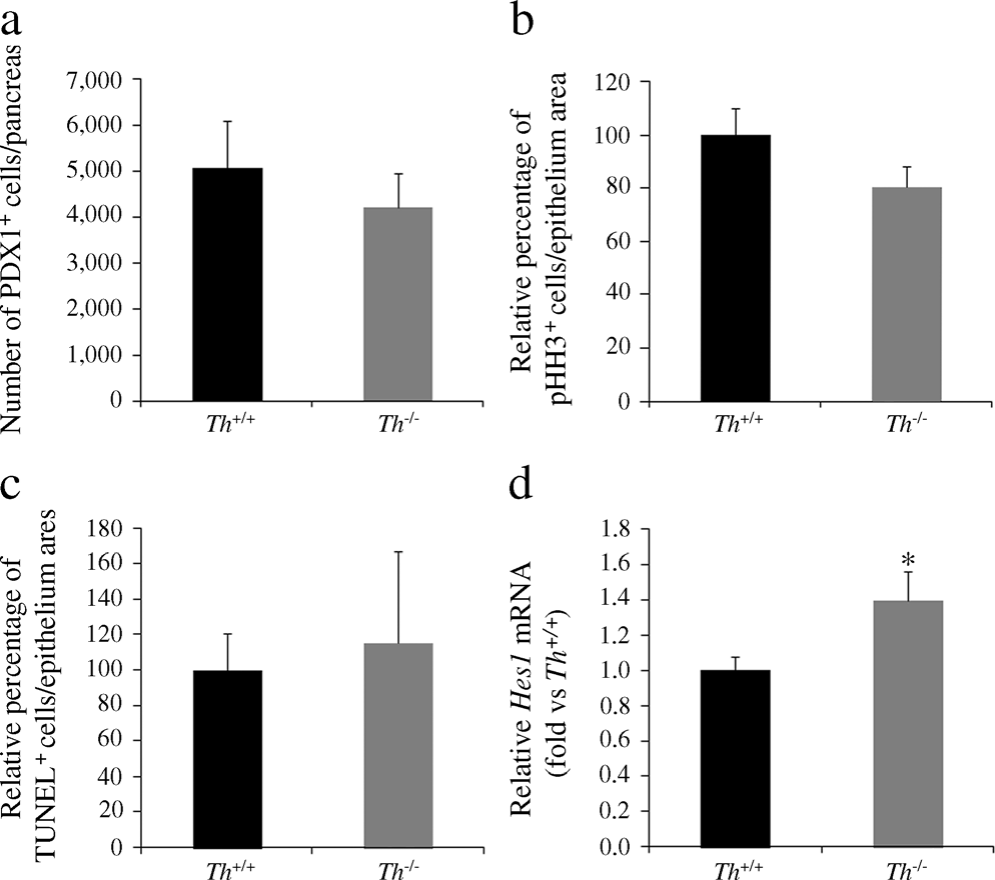
*Hes1* expression is increased in the pancreas of TH-deficient embryos. Quantification of the number of (**a**) PDX1- or (**b**) phospho-histone H3 (pHH3)-expressing cells in sections of E12.5 pancreas. Results represent the mean ± SEM of five whole pancreases for each genotype. (**c**) Quantification of the number of TUNEL^+^ cells in the pancreatic epithelial area at E13.5. Results represent the mean ± SEM of three whole pancreases for each genotype. (**d**) Quantitative real-time PCR of individual pancreases of E12.5 embryos. The levels of *Hes1* transcript were normalised to 18S rRNA and *Th* ^*+/+*^ values were set at 1. Results represent the mean ± SEM of at least seven pancreases per genotype. *p < 0.05 vs *Th* ^*+/+*^

We investigated whether the decrease in the number of NGN3^+^ endocrine precursors resulted from depletion of the pancreatic progenitor pool. Immunostaining with the progenitor marker PDX1 revealed comparable pancreatic progenitor populations in wild-type and TH-deficient embryos (Fig. 5a). This finding is in agreement with the absence of changes seen in the E-cadherin^+^ epithelial area (data not shown). Moreover, the pancreatic epithelium proliferation rate, as measured using the mitotic marker phospho-histone H3 (pHH3), was similar in both genotypes (Fig. 5b), and we observed no differences in the cell death rate, as measured by TUNEL-staining (Fig. 5c). These results rule out the possibility that alterations in either the rate of pancreatic epithelial proliferation or of cell death mediate the observed decrease in the number of NGN3^+^ precursors. We next focused on the Notch signalling pathway, which negatively regulates *Neurog3* expression through the transcriptional repressor HES1 [20]. As *Neurog3* gene activation requires the release of HES1-mediated repression, we analysed *Hes1* mRNA expression levels and found them significantly higher in the *Th* ^***−****/****−***^ vs *Th* ^*+/+*^ pancreas (Fig. 5d).

### Insulin-expressing cells in pancreas explants are increased by dopamine treatment and decreased by TH inhibition

**Fig. 6:**
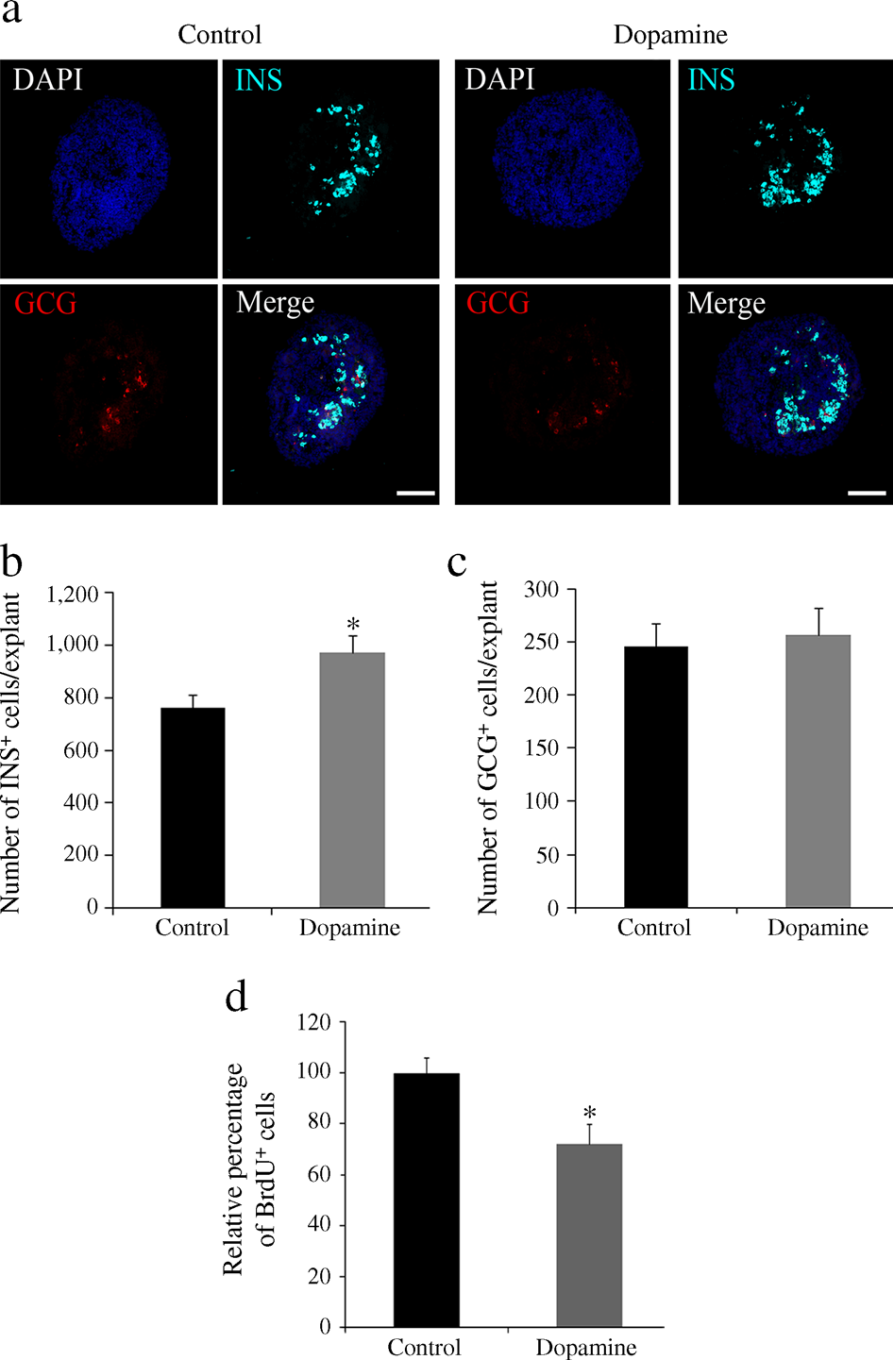
Dopamine administration increases the number of insulin-expressing cells in pancreatic explants. (**a**) Double immunostaining for insulin (INS, cyan) and glucagon (GCG, red) in explants cultured for 5 days. Nuclei are stained with DAPI. Scale bar, 100 μm. (**b**, **c**) Quantification of total number of insulin- and glucagon-expressing cells. Results represent the mean ± SEM of five explants per condition. *p < 0.05 vs control (**d**) Quantification of the number of BrdU^+^ cells in cytospin of explants cultured for 5 days. Results represent the mean ± SEM of at least four explants per condition. *p < 0.05 vs control

Characterisation of the TH-deficient pancreas revealed that catecholamines are important modulators of endocrine and beta cell differentiation. Indeed, analysis of catecholamine content by ELISA confirmed the presence of dopamine (289 fg/pancreas) and noradrenaline (norepinephrine; 652 fg/pancreas) in the E13.5 pancreatic primordium in vivo, as well as in E13.5 explants cultured for 24 h (dopamine, 87.3 fg/explant; noradrenaline, 290 fg/explant). We next investigated the pro-beta cell potential of catecholamines in vitro, treating pancreas explants with dopamine (the initial catecholamine of the pathway). We had previously confirmed the presence of dopamine receptors by quantitative real-time PCR. All five receptors tested were present by E11.5 and were expressed with distinct developmental profiles (ESM Fig. 4). Beginning at E13.5 (day 0), explants were treated daily for 5 days with dopamine. The total number of insulin^+^ cells was higher in dopamine-treated vs untreated explants (Fig. 6a, b) but, in agreement with our in vivo observations, we detected no change in the total number of glucagon-expressing cells (Fig. 6a, c). The proliferation rate at 2 days post culture, as measured by BrdU incorporation, was unaffected by dopamine treatment (data not shown), but decreased after 5 days of culture (Fig. 6d). Furthermore, analysis of the double-labelled insulin/BrdU cells showed that dopamine did not affect the extremely low proliferation rate of the differentiated insulin^+^ cells (data not shown).

In contrast to the pro-beta cell effect of dopamine, challenging pancreatic explants for 1 day with an inhibitor of TH (α-methyl-l-tyrosine) decreased their insulin content relative to that in the controls (ESM Fig. 5a, b).

## Discussion

The postnatal number of pancreatic islet cells depends on the size of the progenitor population, its rate of differentiation and the rates of proliferation and cell death of progenitors/precursors and mature endocrine cells. Here, we show that TH expression in the developing pancreas is required to generate the necessary number of endocrine NGN3^+^ precursors and insulin^+^ cells via a mechanism involving changes in the expression of the transcription factor *Hes1*.

We confirm and build upon previously published findings [22, 32–34] demonstrating TH expression in a small subpopulation of early glucagon^+^ cells, which appear as early as E9.5 in the mouse pancreatic bud. Early glucagon^+^ cells form a distinctive endocrine cell pool that does not express the complement of markers typical of mature glucagon-secreting cells [35]. Our results indicate that TH expression is another distinct feature of a discrete subpopulation of these heterogeneous early glucagon^+^ cells.

Transient catecholamine biosynthetic enzyme expression has been described in a number of embryonic neuronal subpopulations during development [36, 37], including cells found in the stomach and duodenum [36] that express both TH and neurofilament proteins. However, the pancreatic early catecholaminergic cells described here are, to the best of our knowledge, the only embryonic cell type with a non-neuronal endocrine phenotype, as evidenced by the lack of βIII-tubulin, neurofilament, SOX10 or PHOX2B expression. Interestingly, we observed a certain degree of overlap between some of the transcription factors involved in alpha cell specification and subsequent glucagon expression, and the set of transcription factors active in catecholaminergic neurons, i.e. aristaless-related homeobox (ARX), forkhead box protein A1 (FOXA1), FOXA2 [38–40]. Significant parallels have been described between the transcription factor networks involved in the formation of beta cells and those found in serotonergic neurons [41]. However, the molecular mediators of TH transcriptional activation in the subpopulation of TH^+^/glucagon^+^ cells remains unknown.

We provide novel data on the functional significance of the TH-catecholamine pathway during development. TH expression, although restricted to a small proportion of endocrine cells is crucial for the initiation (at E13.5) of the massive wave of beta cell differentiation. At this stage, the number of insulin^+^ cells in the *Th* ^***−****/****−***^ embryonic pancreas was reduced and the relevance of this initial decrease is supported by the lower number of insulin^+^ cells in the *Th* ^***−****/****−***^ pancreatic explants after 5 days in culture. The observed decrease in insulin^+^ cells at E13.5 was accompanied by a decrease in the pool of endocrine precursors expressing NGN3, and in the number of precursors expressing its downstream target gene *Nkx2.2*. NGN3 triggers the activation of a set of transcription factors, including NKX2.2, that are involved in driving the differentiation of insulin-producing beta cells [42]. However, in *Th* ^***−****/****−***^ embryos the general pool of PDX1^+^ pancreatic progenitors and the pancreatic epithelial area were comparable with those in wild-type embryos, in agreement with the absence of changes in proliferation and the apoptotic index, suggesting that the TH deficiency affects beta cell neogenesis.

Although it has been shown that NCCs exert an important influence in beta cell differentiation [43], two observations argue against an implication of NCCs in the TH-deficiency-induced beta cell phenotype: (1) TH is not co-expressed with either SOX10 or PHOX2B; (2) expression of *Sox10* and *Phox2b* is not affected by *Th* deletion (ESM Fig. 3).

No changes in the number of alpha cells (the other differentiating endocrine cells found at the same developmental time-point) were observed in E13.5 *Th* ^***−***/***−***^ embryos or in pancreatic explants after 5 days of dopamine treatment. A selective effect on beta cell differentiation has also been described for retinoic acid [44]. It is unclear why the decrease in the NGN3^+^ and NKX2.2^+^ progenitor pools observed in TH-deficient mice does not affect the number of alpha cells. We can only speculate that catecholamines play a more central role in the triggering or maintenance of beta cell differentiation than of alpha-cell differentiation, or in the induction of distinct classes of NGN3^+^ progenitors that preferentially differentiate into insulin cells.

It has been shown that the competence of the pancreatic epithelium to generate the different endocrine cell types upon NGN3 expression changes throughout development, suggesting the existence of heterogeneous pools of progenitors and/or different extrinsic cues during pancreas organogenesis [45].

Given that TH expression promotes the beta cell differentiation programme, as evidenced by the pancreatic phenotype of the TH-deficient mouse, we studied the pro-beta cell effect of catecholamines in vitro. Whereas inhibition of TH activity in pancreatic explants decreased insulin levels, the exogenous addition of dopamine increased the number of insulin-producing cells without affecting the number of glucagon^+^ cells. This effect occurred in the absence of increased proliferation. Moreover, pancreas cultured with dopamine exhibited a lower proliferation rate at the end of the culture period. This could be due to exhaustion of the progenitor pool as a result of the activation of the differentiation programme.

Taking into consideration that both dopamine and noradrenaline are present endogenously in the E13.5 pancreatic primordium, and that dopamine and adrenergic receptors (data not shown) are present in the embryonic pancreas, it is not possible to conclude which catecholamine is the main player mediating the pro-beta cell effect. In fact, dopamine added exogenously could be converted into noradrenaline. Additional studies are required to further explore these possibilities.

We next investigated the possible involvement of Notch pathway in order to identify the mechanism at work upstream of NGN3. During the initial steps of pancreas organogenesis, Notch signalling is required to maintain an adequate progenitor pool, thus precluding premature differentiation. This is achieved by the expression of the Notch effector *Hes1,* which directly inhibits *Neurog3* transcriptional activity; triggering of the endocrine programme is dependent on the release of *Neurog3* from the inhibitory influence of HES1 [46]. In the pancreas of *Th* ^***−****/****−***^ embryos, in contrast with *Th* ^*+/+*^ mice, the decrease in NGN3 expression was accompanied by an increase in *Hes1* expression, placing the catecholamine effect, at least in part, at the level of Notch signalling regulation.

We did not find a decrease in *Hes1* expression in E13.5 cultured pancreas explants after 1, 2 or 5 days of dopamine treatment (data not shown). This apparent discrepancy between the in vivo and in vitro results could be due to the different experimental conditions (with lack of additional signals from the surrounding tissues in the case of the explants), or to the different developmental stages of *Hes1* analysis—primary transition (E12.5) in vivo and secondary transition (post E13.5) in cultured explants. We favour this second possibility since recent studies propose that Notch signalling may have two distinct roles in pancreas development, depending on the stage: (1) an early role preventing premature precursor differentiation and, later, (2) an effect on lineage specification [47]. Nonetheless, our explant experiments suggest that dopamine, after initiation of the secondary transition, may be acting through an additional mechanism.

Catecholamines are molecules with well-described effects on adult islet cell secretion and may be involved in islet cell adaptation in insulin resistance [48]. The size of mature islets and the number of insulin-producing cells they contain are critical determinants of pancreatic postnatal function and the risk of developing diabetes [17, 49]. Therefore, our identification of TH/catecholamines as modulators of the generation of insulin-producing cells during development, or unravelling in the future further actions of this pathway upon endocrine maturation, may have important implications for strategies aimed at regenerating or replacing beta cells in patients with diabetes.

Importantly, the results presented here are consistent with the paradigm whereby molecules that act as intercellular signalling mediators, with well-defined restricted roles in postnatal organisms, are present at embryonic stages, during which they participate in diverse functions often unrelated to their later roles [50].

## Electronic supplementary material

Below is the link to the electronic supplementary material.

ESM Methods (PDF 57.3 kb)

ESM Fig. 1 (PDF 32.7 kb)

ESM Fig. 2 (PDF 359 kb)

ESM Fig. 3 (PDF 93.4 kb)

ESM Fig. 4 (PDF 132 kb)

ESM Fig. 5 (PDF 293 kb)

ESM Table 1 (PDF 73.6 kb)

ESM Table 2 (PDF 11.4 kb)

ESM Table 3 (PDF 22.7 kb)

ESM Table 4 (PDF 28 kb)

## Abbreviations

E: Embryonic day
NCC: Neural crest cell
NGN3: Neurogenin 3
NKX2.2: NK2 homeobox 2
PDX1: Pancreatic and duodenal homeobox 1
PHOX2b: Paired-like homeobox 2b
PTF1A: Pancreas transcription factor 1 complex
SOX10: SRY (sex determining region Y)-box 10

## References

[1] Collombat P, Xu X, Heimberg H, Mansouri A (2010). Pancreatic beta-cells: from generation to regeneration. Semin Cell Dev Biol.

[2] Kroon E, Martinson LA, Kadoya K (2008). Pancreatic endoderm derived from human embryonic stem cells generates glucose-responsive insulin-secreting cells in vivo. Nat Biotechnol.

[3] Arda HE, Benitez CM, Kim SK (2013). Gene regulatory networks governing pancreas development. Dev Cell.

[4] Oliver-Krasinski JM, Stoffers DA (2008). On the origin of the beta cell. Genes Dev.

[5] Gittes GK (2009). Developmental biology of the pancreas: a comprehensive review. Dev Biol.

[6] Heinis M, Simon MT, Duvillie B (2010). New insights into endocrine pancreatic development: the role of environmental factors. Horm Res Paediatr.

[7] Kim SK, Hebrok M (2001). Intercellular signals regulating pancreas development and function. Genes Dev.

[8] Ahlgren U, Jonsson J, Edlund H (1996). The morphogenesis of the pancreatic mesenchyme is uncoupled from that of the pancreatic epithelium in IPF1/PDX1-deficient mice. Development.

[9] Guz Y, Montminy MR, Stein R (1995). Expression of murine STF-1, a putative insulin gene transcription factor, in beta cells of pancreas, duodenal epithelium and pancreatic exocrine and endocrine progenitors during ontogeny. Development.

[10] Kawaguchi Y, Cooper B, Gannon M, Ray M, MacDonald RJ, Wright CV (2002). The role of the transcriptional regulator Ptf1a in converting intestinal to pancreatic progenitors. Nat Genet.

[11] Pan FC, Wright C (2011). Pancreas organogenesis: from bud to plexus to gland. Dev Dyn.

[12] Attali M, Stetsyuk V, Basmaciogullari A (2007). Control of beta-cell differentiation by the pancreatic mesenchyme. Diabetes.

[13] Jorgensen MC, Ahnfelt-Ronne J, Hald J, Madsen OD, Serup P, Hecksher-Sorensen J (2007). An illustrated review of early pancreas development in the mouse. Endocr Rev.

[14] Cano DA, Soria B, Martin F, Rojas A (2014). Transcriptional control of mammalian pancreas organogenesis. Cell Mol Life Sci.

[15] Gradwohl G, Dierich A, LeMeur M, Guillemot F (2000). Neurogenin3 is required for the development of the four endocrine cell lineages of the pancreas. Proc Natl Acad Sci U S A.

[16] Gu G, Dubauskaite J, Melton DA (2002). Direct evidence for the pancreatic lineage: NGN3^+^ cells are islet progenitors and are distinct from duct progenitors. Development.

[17] Sussel L, Kalamaras J, Hartigan-O’Connor DJ (1998). Mice lacking the homeodomain transcription factor Nkx2.2 have diabetes due to arrested differentiation of pancreatic beta cells. Development.

[18] Apelqvist A, Li H, Sommer L (1999). Notch signalling controls pancreatic cell differentiation. Nature.

[19] Jensen J, Heller RS, Funder-Nielsen T (2000). Independent development of pancreatic alpha- and beta-cells from neurogenin3-expressing precursors: a role for the notch pathway in repression of premature differentiation. Diabetes.

[20] Jensen J, Pedersen EE, Galante P (2000). Control of endodermal endocrine development by Hes-1. Nat Genet.

[21] Lee JC, Smith SB, Watada H (2001). Regulation of the pancreatic pro-endocrine gene neurogenin3. Diabetes.

[22] Teitelman G, Lee JK (1987). Cell lineage analysis of pancreatic islet development: glucagon and insulin cells arise from catecholaminergic precursors present in the pancreatic duct. Dev Biol.

[23] O’Keeffe GC, Barker RA, Caldwell MA (2009). Dopaminergic modulation of neurogenesis in the subventricular zone of the adult brain. Cell Cycle.

[24] Fitch SR, Kimber GM, Wilson NK (2012). Signaling from the sympathetic nervous system regulates hematopoietic stem cell emergence during embryogenesis. Cell Stem Cell.

[25] Lopez-Sanchez C, Bartulos O, Martinez-Campos E (2010). Tyrosine hydroxylase is expressed during early heart development and is required for cardiac chamber formation. Cardiovasc Res.

[26] Zhou QY, Quaife CJ, Palmiter RD (1995). Targeted disruption of the tyrosine hydroxylase gene reveals that catecholamines are required for mouse fetal development. Nature.

[27] Gouzi M, Kim YH, Katsumoto K, Johansson K, Grapin-Botton A (2011). Neurogenin3 initiates stepwise delamination of differentiating endocrine cells during pancreas development. Dev Dyn.

[28] Herrera PL (2000). Adult insulin- and glucagon-producing cells differentiate from two independent cell lineages. Development.

[29] Herrera PL, Huarte J, Zufferey R (1994). Ablation of islet endocrine cells by targeted expression of hormone-promoter-driven toxigenes. Proc Natl Acad Sci U S A.

[30] Desgraz R, Herrera PL (2009). Pancreatic neurogenin 3-expressing cells are unipotent islet precursors. Development.

[31] Watada H, Scheel DW, Leung J, German MS (2003). Distinct gene expression programs function in progenitor and mature islet cells. J Biol Chem.

[32] Teitelman G, Joh TH, Reis DJ (1981). Linkage of the brain-skin-gut axis: islet cells originate from dopaminergic precursors. Peptides.

[33] Teitelman G, Joh TH, Reis DJ (1981). Transformation of catecholaminergic precursors into glucagon (A) cells in mouse embryonic pancreas. Proc Natl Acad Sci U S A.

[34] Teitelman G, Alpert S, Polak JM, Martinez A, Hanahan D (1993). Precursor cells of mouse endocrine pancreas coexpress insulin, glucagon and the neuronal proteins tyrosine hydroxylase and neuropeptide Y, but not pancreatic polypeptide. Development.

[35] Wilson ME, Kalamaras JA, German MS (2002). Expression pattern of IAPP and prohormone convertase 1/3 reveals a distinctive set of endocrine cells in the embryonic pancreas. Mech Dev.

[36] Baetge G, Gershon MD (1989). Transient catecholaminergic (TC) cells in the vagus nerves and bowel of fetal mice: relationship to the development of enteric neurons. Dev Biol.

[37] Jonakait GM, Markey KA, Goldstein M, Black IB (1984). Transient expression of selected catecholaminergic traits in cranial sensory and dorsal root ganglia of the embryonic rat. Dev Biol.

[38] Bramswig NC, Kaestner KH (2011). Transcriptional regulation of alpha-cell differentiation. Diabetes Obes Metab.

[39] Filippi A, Jainok C, Driever W (2012). Analysis of transcriptional codes for zebrafish dopaminergic neurons reveals essential functions of Arx and Isl1 in prethalamic dopaminergic neuron development. Dev Biol.

[40] Stott SR, Metzakopian E, Lin W, Kaestner KH, Hen R, Ang SL (2013). Foxa1 and foxa2 are required for the maintenance of dopaminergic properties in ventral midbrain neurons at late embryonic stages. J Neurosci.

[41] Ohta Y, Kosaka Y, Kishimoto N (2011). Convergence of the insulin and serotonin programs in the pancreatic beta-cell. Diabetes.

[42] Rukstalis JM, Habener JF (2009). Neurogenin3: a master regulator of pancreatic islet differentiation and regeneration. Islets.

[43] Nekrep N, Wang J, Miyatsuka T, German MS (2008). Signals from the neural crest regulate beta-cell mass in the pancreas. Development.

[44] Ostrom M, Loffler KA, Edfalk S (2008). Retinoic acid promotes the generation of pancreatic endocrine progenitor cells and their further differentiation into beta-cells. PLoS One.

[45] Johansson KA, Dursun U, Jordan N (2007). Temporal control of neurogenin3 activity in pancreas progenitors reveals competence windows for the generation of different endocrine cell types. Dev Cell.

[46] Shih HP, Kopp JL, Sandhu M (2012). A Notch-dependent molecular circuitry initiates pancreatic endocrine and ductal cell differentiation. Development.

[47] Afelik S, Qu X, Hasrouni E (2012). Notch-mediated patterning and cell fate allocation of pancreatic progenitor cells. Development.

[48] Ahren B (2000). Autonomic regulation of islet hormone secretion—implications for health and disease. Diabetologia.

[49] Costes S, Langen R, Gurlo T, Matveyenko AV, Butler PC (2013). β-Cell failure in type 2 diabetes: a case of asking too much of too few?. Diabetes.

[50] Hernandez-Sanchez C, Mansilla A, de la Rosa EJ, de Pablo F (2006). Proinsulin in development: new roles for an ancient prohormone. Diabetologia.

