# Supplementary material for: Non-neural tyrosine hydroxylase, via modulation of endocrine pancreatic precursors, is required for normal development of beta cells in the mouse pancreas
Source: Diabetologia. 2014 Aug 1;57(11):2339–47. doi: 10.1007/s00125-014-3341-6 (PMC4181516; doi:10.1007/s00125-014-3341-6)
Supplement: Supplementary file 1 — (PDF 57.3 kb) [file 125_2014_3341_MOESM1_ESM.pdf]

Electronic Supplementary Material

Materials and Methods

Primer and probe sequences, and antibodies used are provided in ESM Tables 1-3. The sources of chemical substances, equipment and software are provided in ESM Table 4.

Mice and embryos

The morning on which the vaginal plug was observed was designated embryonic day 0.5 (E0.5). Pregnant mice were sacrificed by cervical dislocation on different days of gestation and the embryos removed.

Immunoblotting

At the earliest time points (E11.5, E12.5 and E13.5), five isolated pancreatic buds were combined. At later stages (E14.5-E15.5), pools of three buds were used. Individual pancreases were used in the analysis of $Th^{+/+}$ vs $Th^{-/-}$ genotypes. Tissues were homogenised for 15 min at 4ºC in lysis buffer (in mmol/L: 50 HEPES, 100 NaCl, 10 EDTA, 1 orthovanadate, 25 NaF, 4 sodium pyrophosphate) containing 1% (wt/vol) Triton x-100 and a mini EDTA-free protease inhibitor tablet. Cell lysates were centrifuged at 20,000g, at 4ºC for 10 min, and the supernatants were collected and stored at -20ºC until further analysis. For immunoblotting, 6 µg of pancreatic protein extract or 0.6 µg of adrenal gland protein extract were fractionated by SDS-PAGE electrophoresis in 12% (wt/vol) polyacrylamide gels and transferred to nitrocellulose membranes. Blots were blocked in 5% (wt/vol) BSA in PBS-Tween-20 (0.05% wt/vol) and incubated overnight at 4ºC with anti-TH antibody (1:500). After stripping, the membranes were re-blotted with anti-β-actin or tubulin as loading controls. Antibodies were detected with the corresponding horseradish peroxidase-labeled secondary antibodies, and visualised with the Super Signal West Pico chemiluminescent substrate.

Image analysis

Images were collected by confocal microscopy (Leica TCS-SP5). For morphometric analysis, quantification of the total and epithelial pancreatic area was performed in E-cadherin-stained sections using the Image J 1.48v (http://imagej.nih.gov/ij). The number of cells expressing a specific marker was performed as follows. The total number of glucagon, insulin, PDX1, NGN3, and NKX2.2-expressing cells, was determined by quantifying all positive cells per section in all sections for each pancreas. Phospho-histone H3 (pHH3) and TUNEL-positive cells were counted throughout the whole E-cadherin-labeled pancreatic epithelium. Data were normalised to the E-cadherin-positive area and expressed relative to the wild-type pancreas.

The insulin pixels analysis in explants was performed in whole pancreases using Image J 1.48v software. For quantification all photos were acquired under the same conditions of laser intensity and gain, and the pixels corresponding to insulin (cyan color) were quantified. The data are relative to the whole explant.

Immunohistochemistry

For immunohistochemical analysis, sections of E12.5 and E13.5 embryos or cultured explants were fixed overnight at 4ºC in 4% (wt/vol) PFA, then rinsed with PBS and dehydrated through an ethanol gradient before embedding in paraffin for microtome sectioning. Paraffin sections (7-8 μm) were de-waxed in Histo-Clear-II and rehydrated through a descending series of ethanol dilutions. Antigen retrieval was achieved by microwaving [twice for 5 min at 600 W in 0.01 mmol/l citrate buffer (pH 6) with 0.05% (wt/vol) Tween-20] followed by three washes in PBS. Tissue sections were permeabilised with PBS-Triton X-100 (1% wt/vol). Non-specific binding was blocked with 3% (vol/vol) donkey immunoserum in PBS-Triton X-100 (1% w/v). Sections were

incubated overnight at 4ºC with primary antibodies in blocking buffer, and then incubated with secondary antibodies labeled with either Alexa-488, Alexa-568 or Alexa-647 dyes). Nuclei were labeled with DAPI. For whole mount immunohistochemistry, pancreas explants were fixed in 4% (wt/vol) PFA and permeabilised with PBS-Triton X-100 (2% wt/vol); immunochemistry was carried out as described above for sections.

BrdU detection and cell counting in cytospin

For cell proliferation experiments, explants were treated with 5 μmol/L BrdU for one or two hours at the end of the culture period. Cultured pancreases were individually collected and the cells dissociated by treatment at 37ºC for 5 min with 2 g/L trypsin in PBS/BSA (3 g/L), followed by mechanical disaggregation. Dissociated cells were fixed for at least 2 hours with 4% (wt/vol) PFA, washed with PBS and then stored in PBS/BSA (3 g/L) at 4ºC until immunohistochemistry was performed. Cells were cyto-centrifuged on poly-l-lysin-pretreated glass slides for 5 min at 700 rpm, permeabilised with PBS-Triton X-100 (0.4% wt/vol) for 20 min and treated with HCl 2N (10 min) followed by sodium borate (0.1 mmol/L) (pH 8.9) for 10 min. Immunohistochemistry was performed as described above.

ELISA

Catecholamines were determined by ELISA (3-CAT research ELISA, BA E-5600). A pool of 46 pancreatic primordia at E13.5 and a pool of 43 E13.5 pancreatic explants cultured for 24h were homogenised, and catecholamines extracted and measured following the manufacturer instructions.
