# Supplementary material for: Non-neural tyrosine hydroxylase, via modulation of endocrine pancreatic precursors, is required for normal development of beta cells in the mouse pancreas
Source: Diabetologia. 2014 Aug 1;57(11):2339–47. doi: 10.1007/s00125-014-3341-6 (PMC4181516; doi:10.1007/s00125-014-3341-6)
Supplement: Supplementary file 2 — (PDF 32.7 kb) [file 125_2014_3341_MOESM2_ESM.pdf]

ESM Fig.1

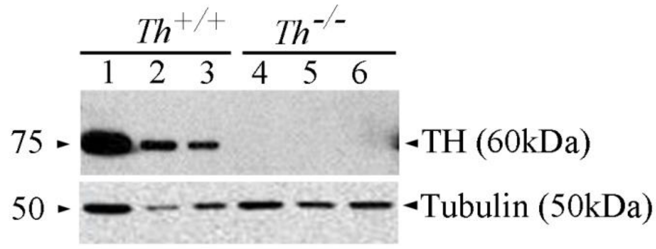

TH-immunoblot of *Th*$^{+/+}$ and *Th*$^{-/-}$ pancreases. TH-Immunoblot of protein extracts of individual pancreases of *Th*$^{+/+}$ and *Th*$^{-/-}$ embryos (E12.5). Tubulin was used as a loading control. TH protein was not detected in *Th*$^{-/-}$ pancreases. This result confirms the genotype of the embryos and the specificity of the antibody.
