# Supplementary material for: Non-neural tyrosine hydroxylase, via modulation of endocrine pancreatic precursors, is required for normal development of beta cells in the mouse pancreas
Source: Diabetologia. 2014 Aug 1;57(11):2339–47. doi: 10.1007/s00125-014-3341-6 (PMC4181516; doi:10.1007/s00125-014-3341-6)
Supplement: Supplementary file 3 — (PDF 359 kb) [file 125_2014_3341_MOESM3_ESM.pdf]

ESM Fig. 2

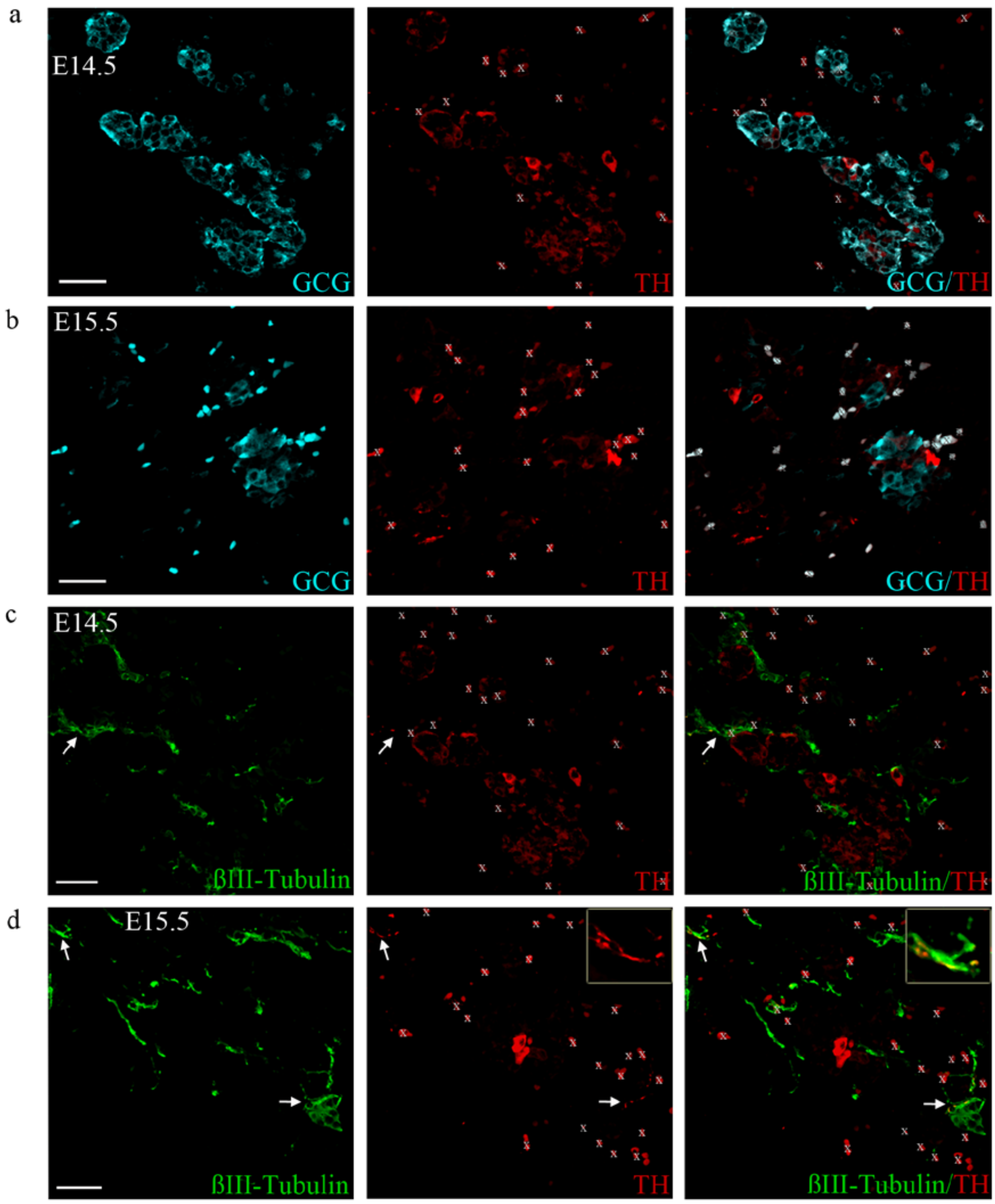

Characterisation of the TH$^+$cells at E14.5 and E15.5. Double-immunostaining for glucagon (GCG, cyan) and TH-mouse (red) **(a, b)** or βIII-tubulin (green) and TH-mouse (red) **(c, d)** in pancreatic sections of E14.5 **(a, c)** and E15.5 **(b, d)** embryos. Arrows show sympathetic neuronal projections (TH$^+$ and βIII–Tubulin$^+$). White-X indicates blood cells. Insets show a magnification of representative cells. Scale bar, 50 µm.
