# Supplementary material for: Non-neural tyrosine hydroxylase, via modulation of endocrine pancreatic precursors, is required for normal development of beta cells in the mouse pancreas
Source: Diabetologia. 2014 Aug 1;57(11):2339–47. doi: 10.1007/s00125-014-3341-6 (PMC4181516; doi:10.1007/s00125-014-3341-6)
Supplement: Supplementary file 4 — (PDF 93.4 kb) [file 125_2014_3341_MOESM4_ESM.pdf]

ESM Fig. 3

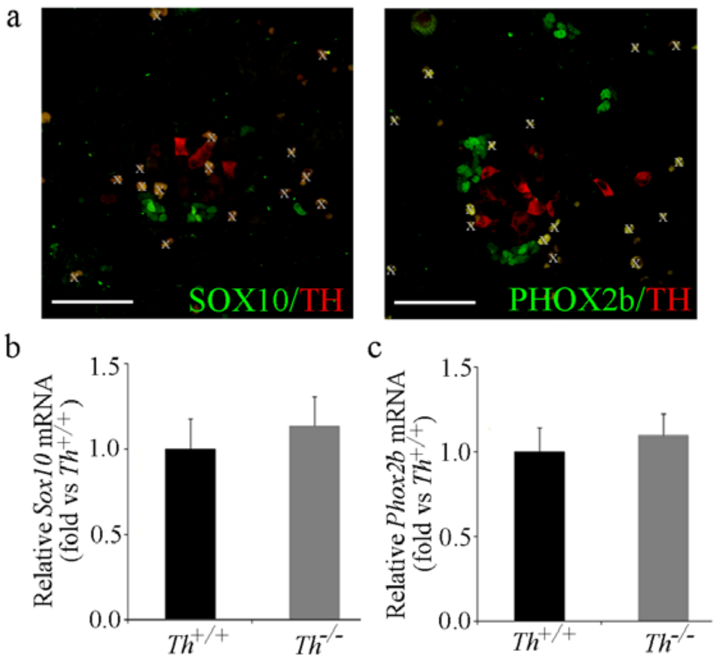

SOX10 and PHOX2B do not co-express with TH and their expression is not affected by *Th* deletion. **(a)** Co-Immunostaining for SOX10 (green) or PHOX2b (green) and TH-mouse (red) in pancreatic sections of E13.5. White-X indicates blood cells. Scale bar, 50 μm. **(b, c)** Quantitative real-time PCR of individual pancreases at E12.5. The levels of *Sox10* and *Phox2b* transcripts were normalised to 18S rRNA and $Th^{+/+}$ values were set at 1. Results represent the mean ± SEM of at least ten pancreases per genotype.
