# Supplementary material for: Non-neural tyrosine hydroxylase, via modulation of endocrine pancreatic precursors, is required for normal development of beta cells in the mouse pancreas
Source: Diabetologia. 2014 Aug 1;57(11):2339–47. doi: 10.1007/s00125-014-3341-6 (PMC4181516; doi:10.1007/s00125-014-3341-6)
Supplement: Supplementary file 5 — (PDF 132 kb) [file 125_2014_3341_MOESM5_ESM.pdf]

ESM Fig. 4

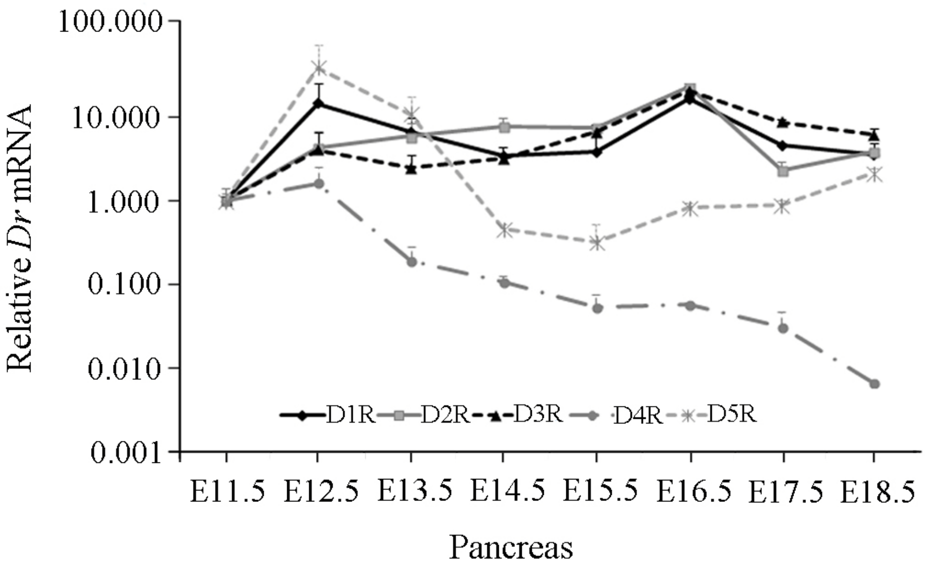

Relative expression of dopamine receptors during pancreas development. Analysis of dopamine receptors (*D1r-D5r*) gene expression performed by quantitative real-time PCR of three to five pooled pancreases from E11.5 to E18.5 embryos. Levels of each dopamine receptor transcript were normalised to 18S rRNA and E11.5 values were set at 1. Data are presented on a logarithmic scale. Results represent the mean ± SEM of at least three different pools of each embryonic age.
