# Supplementary material for: Non-neural tyrosine hydroxylase, via modulation of endocrine pancreatic precursors, is required for normal development of beta cells in the mouse pancreas
Source: Diabetologia. 2014 Aug 1;57(11):2339–47. doi: 10.1007/s00125-014-3341-6 (PMC4181516; doi:10.1007/s00125-014-3341-6)
Supplement: Supplementary file 6 — (PDF 293 kb) [file 125_2014_3341_MOESM6_ESM.pdf]

ESM Fig. 5

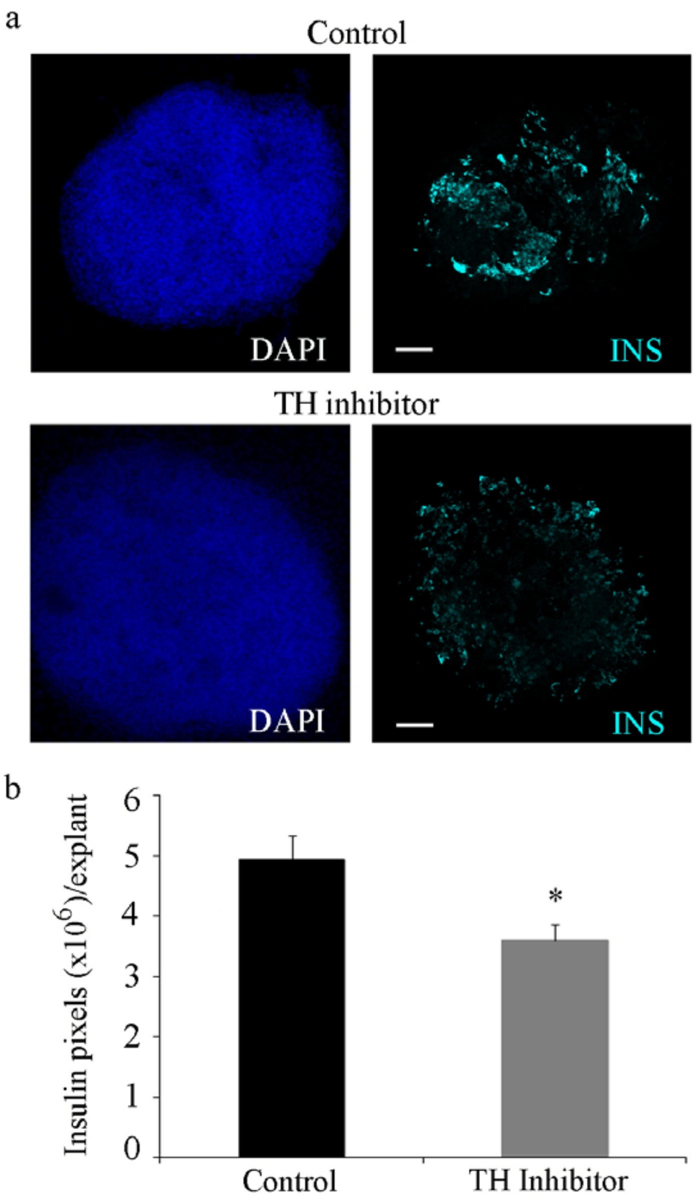

TH inhibition decreases the insulin content of pancreatic explants in culture.

(a) Immunostaining for insulin (INS, cyan) in pancreatic explants cultured in the absence or presence of TH inhibitor for 1 day. Nuclei are stained with DAPI. Scale bar, 100 μm. **(b)** Quantification of the total insulin pixels of immunofluorescence relative to the explant. Results represent the mean $\pm$ SEM of at least seven explants per treatment. *$p<0.05$ vs control.
