# Supplementary material for: Non-neural tyrosine hydroxylase, via modulation of endocrine pancreatic precursors, is required for normal development of beta cells in the mouse pancreas
Source: Diabetologia. 2014 Aug 1;57(11):2339–47. doi: 10.1007/s00125-014-3341-6 (PMC4181516; doi:10.1007/s00125-014-3341-6)
Supplement: Supplementary file 7 — (PDF 73.6 kb) [file 125_2014_3341_MOESM7_ESM.pdf]

ESM Table 1. Sequence of the primers used in quantitative real-time PCR with the indicated probes from the Universal Probe Library (UPL).

| Gene | Primer Forward | Primer Reverse | UPL probe | Amplicon size (base pairs) |
|---|---|---|---|---|
| *Th* | GGAACGGTACTGTGGCTACC | CCTTCAAGAAGTGAGACACATCC | 56 | 68 |
| *D1r* | AGATGCCGAGGATGACAACT | GGGAGGATGAAATGGCGTA | 15 | 62 |
| *D2r* | GTCAACACCAAGCGTAGCAG | TTGCCCTTGAGTGGTGTCTT | 21 | 62 |
| *D3r* | CACGTGTCCCCAGAGCTT | TCAGGGCACTGTTCACGTAG | 21 | 61 |
| *D4r* | GGTCTACTCGTCCGTCTGCT | GGCCCAGTAAAGCAGTAGCAT | 4 | 64 |
| *D5r* | CTACCGCATTGCATTGCACAGGTT | CATGCTCAGCTGCCCTTT | 52 | 62 |
| *18S* | TGCGAGTACTCAACACCAACA | TTCCTCAACACCACATGAGC | 70 | 103 |
