# Supplementary material for: Non-neural tyrosine hydroxylase, via modulation of endocrine pancreatic precursors, is required for normal development of beta cells in the mouse pancreas
Source: Diabetologia. 2014 Aug 1;57(11):2339–47. doi: 10.1007/s00125-014-3341-6 (PMC4181516; doi:10.1007/s00125-014-3341-6)
Supplement: Supplementary file 8 — (PDF 11.4 kb) [file 125_2014_3341_MOESM8_ESM.pdf]

ESM Table 2. Taqman assays used in quantitative real-time PCR.

| **Gene** | **Taqman Probe** |
|---|---|
| *Th* | Mm00447557_m1 |
| *Ins1* | Mm01950294_s1 |
| *Ins2* | Mm00731595_gH |
| *Ngn3* | Mm00437606_s1 |
| *Gcg* | Mm01269055_m1 |
| *Nkx2.2* | Mm00839794_m1 |
| *Hes1* | Mm01342805_m1 |
| *Sox10* | Mm01300162_m1 |
| *Phox2b* | Mm00435872_m1 |
| *18S* | Hs99999901_s1 |
