# Supplementary material for: Non-neural tyrosine hydroxylase, via modulation of endocrine pancreatic precursors, is required for normal development of beta cells in the mouse pancreas
Source: Diabetologia. 2014 Aug 1;57(11):2339–47. doi: 10.1007/s00125-014-3341-6 (PMC4181516; doi:10.1007/s00125-014-3341-6)
Supplement: Supplementary file 9 — (PDF 22.7 kb) [file 125_2014_3341_MOESM9_ESM.pdf]

ESM Table 3. List of antibodies used in immunohistochemistry and western blot experiments.

Primary antibodies	Dilution	Host species	Company	Reference
Anti-insulin	1/200	Guinea pig	Abcam	Ab7842
Anti-glucagon	1/300	Rabbit	DAKO	A0565
Anti-glucagon	1/300	Guinea pig	LINCO Research INC.	4031-01F
Anti-tyrosine hydroxylase	1/100	Rabbit	Millipore	AB152
Anti-tyrosine hydroxylase	1/100	Mouse	Millipore	MAB318
Anti-PDX1	1/20	Mouse	Hybrydoma Bank	F6A11
Anti-NGN3	1/20	Mouse	Hybrydoma Bank	F25A1B3
Anti-NKX2.2	1/20	Mouse	Hybrydoma Bank	745 A5
Anti-BrdU	1/250	Mouse	Hybrydoma Bank	G3G4
Anti-pHH3(Ser10)	1/150	Rabbit	Millipore	06-570
Anti-SOX10	1/20	Goat	Santa cruz	Sc-17342
Anti-E-cadherin	1/300	Mouse	BD Transduction Laboratories	610181
Anti-BIII-Tubulin	1/500	Rabbit	Covance	MRB-435P
Anti-NF (neurofilament)	1/50	Mouse	Hybrydoma Bank	2H3c
Anti-β-actin	1/5000	Mouse	Sigma	A5316
Anti-β-tubulin	1/2000	Mouse	Sigma	T4026
