# Supplementary material for: Non-neural tyrosine hydroxylase, via modulation of endocrine pancreatic precursors, is required for normal development of beta cells in the mouse pancreas
Source: Diabetologia. 2014 Aug 1;57(11):2339–47. doi: 10.1007/s00125-014-3341-6 (PMC4181516; doi:10.1007/s00125-014-3341-6)
Supplement: Supplementary file 10 — (PDF 28 kb) [file 125_2014_3341_MOESM10_ESM.pdf]

ESM Table 4. List of the sources of chemical substances.

**Reactive**	**Company**	**City,Country**
Collagen	Roche	Mannheim, Germany
DMEN	Invitrogen	NY, USA
α-methyl-L-tyrosine	Sigma	Diegem, Belgium
BrdU	Sigma	Diegem, Belgium
Dopamine	Sigma	Diegem, Belgium
HEPES	Sigma	Diegem, Belgium
NaCl	Sigma	Diegem, Belgium
EDTA	Sigma	Diegem, Belgium
Orthovanadate	Sigma	Diegem, Belgium
NaF	Sigma	Diegem, Belgium
Sodium pyrophosphate	Sigma	Diegem, Belgium
Triton X-100	Sigma	Diegem, Belgium
EDTA-free protease inhibitor tablet	Roche	Mannheim, Germany
Trypsin	Worthington Biochemical Corporation	New Jersy, USA
Poly-l-lysin	Sigma	Diegem, Belgium
Nitrocellulose membranes	Bio-Rad	CA, USA
Horseradish peroxidase-labeled secondary antibodies	Pierce	Rockford, IL,USA
Super Signal West Pico chemiluminescent substrate	Pierce	Rockford, IL,USA
Secondary antibodies labeled with Alexa-dyes	Molecular Probes	Carslab, CA, USA
Histo-Clear-II	CONDA	Madrid, SPAIN
Donkey inmunoserum	Sigma	Diegem, Belgium
DAPI	Molecular Probes	Carlsbad, USA
Apoptosis Detection System	Promega	Madison, USA
3-CAT Research ELISA	LDN	Nordhorn, Germany
